# Development of Antifouling Strategies for Marine Applications

**DOI:** 10.3390/microorganisms11061568

**Published:** 2023-06-13

**Authors:** Maria João Romeu, Filipe Mergulhão

**Affiliations:** 1 LEPABE—Laboratory for Process Engineering, Environment, Biotechnology and Energy, Faculty of Engineering, University of Porto, Rua Dr. Roberto Frias, 4200-465 Porto, Portugal; mariaromeu@fe.up.pt; 2 ALiCE—Associate Laboratory in Chemical Engineering, Faculty of Engineering, University of Porto, Rua Dr. Roberto Frias, 4200-465 Porto, Portugal

**Keywords:** biofilms, antifouling surfaces, marine biofouling, marine coatings, antifouling strategies

## Abstract

Marine biofouling is an undeniable challenge for aquatic systems since it is responsible for several environmental and ecological problems and economic losses. Several strategies have been developed to mitigate fouling-related issues in marine environments, including developing marine coatings using nanotechnology and biomimetic models, and incorporating natural compounds, peptides, bacteriophages, or specific enzymes on surfaces. The advantages and limitations of these strategies are discussed in this review, and the development of novel surfaces and coatings is highlighted. The performance of these novel antibiofilm coatings is currently tested by *in vitro* experiments, which should try to mimic real conditions in the best way, and/or by *in situ* tests through the immersion of surfaces in marine environments. Both forms present their advantages and limitations, and these factors should be considered when the performance of a novel marine coating requires evaluation and validation. Despite all the advances and improvements against marine biofouling, progress toward an ideal operational strategy has been slow given the increasingly demanding regulatory requirements. Recent developments in self-polishing copolymers and fouling-release coatings have yielded promising results which set the basis for the development of more efficient and eco-friendly antifouling strategies.

## 1. Introduction

Marine biofilm development is a complex and dynamic process comprising several organisms and interactions, which can be affected by different factors, from surface properties to environmental parameters and microbial content [1,2,3,4]. Indeed, biofilms are a common feature on all aquatic submerged surfaces, contributing to marine biofouling, which is responsible for several detrimental impacts on shipping efficiency, aquaculture industries, equipment corrosion, and maintenance, as well as disturbances in ecosystems [5,6,7]. Since cell adhesion and biofilm formation are primordial steps to macrofouling, the most promising marine biofouling mitigation approach is delaying and controlling microfouling events [8,9].

Even though the schematic conceptual biofilm developmental model based on five stages (reversible attachment of planktonic cells, irreversible attachment, biofilm maturation by the development of microcolonies and high extracellular polymeric substance (EPS) production, maturation of the biofilm, and dispersal/detachment) has been widely generalized to describe all biofilms [10], this model does not necessarily describe the complexity of biofilms in the real world, including industrial, clinical, and natural settings as marine environments. Indeed, this model was recently reviewed by the scientific community, which proposed a most inclusive model involving three major events: aggregation, growth, and disaggregation [11]. Therefore, although no developmental model accurately represents biofilm formation for all microorganisms, numerous *in vitro* systems have been designed to study biofilm formation and development to better mimic real conditions [12,13]. Moreover, some of these *in vitro* studies are posteriorly validated and/or confirmed by *in situ* studies in real marine environments [14]. The advantages and limitations of both study types must be considered when choosing the most appropriate method.

There is a pressing need to develop novel antibiofilm surfaces to manage concerns associated with marine fouling and comply with the increasingly strict and demanding legislation in this area [15,16]. Some of these policies involve banning biocides or antifouling paints due to their high persistence and toxicity on non-target marine organisms [17], as well as providing guidelines for the control and management of ship biofouling to minimize the transfer of invasive aquatic species [18]. Several marine coatings have been developed and tested under *in vitro* and/or *in situ* assays. Advancements in polymer science, nanotechnology, and the progress of innovative surface models inspired by nature are expected to significantly impact the improvement of antifouling methodologies, contributing to the development of a new generation of environmentally friendly marine coatings.

This review aims to briefly collect evidence on the development and concerns of marine biofouling and introduce a brief overview of the current marine antifouling strategies used. The advancement and the impact of different marine coatings on marine biofilm development are addressed, focusing on the importance, advantages, and limitations of *in vitro* and *in situ* studies.

## 2. Marine Biofouling

**Figure 1:**
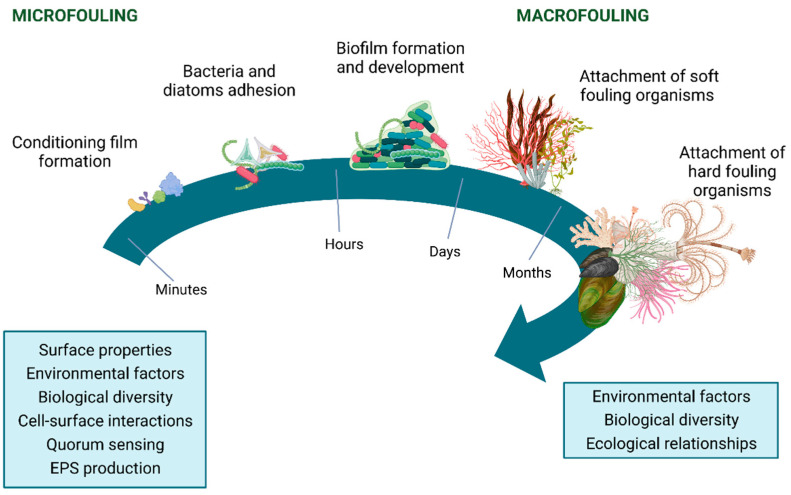
Representation of the marine biofouling process and the main parameters/factors that affect microfouling and macrofouling events. Microfouler organisms include mainly marine bacteria, cyanobacteria, and diatoms, while macrofouler organisms comprise algae, corals, sponges, anemones, tunicates, hydroids, and additional marine invertebrates (soft macrofouler organisms), as well as barnacles, mussels, bryozoans, and tuberworms (hard macrofouler organisms). This image was created with the software BioRender (https://biorender.com/).

Marine biofouling is a dynamic natural process that comprises both microfouling and macrofouling events. Although the diversity and prevalence of fouling organisms depend on geographic location, seasonal variations, and different interactions [19], microfouling includes forming a conditioning film over the submerged surface, the adhesion of microfouler organisms (mainly bacteria, cyanobacteria, and diatoms), followed by biofilm development. In turn, macrofouling implies the attachment and settlement of soft fouler organisms, such as algae, corals, sponges, anemones, tunicates, hydroids, and additional marine invertebrates (e.g., larvae of brine shrimp), as well as barnacles, mussels, bryozoans, and tubeworms (hard fouler organisms) (Figure 1) [19,20].

After the first minutes of immersion, the physicochemical properties of the submerged surface may be modified by the formation of a film comprised of inorganic and organic molecules from the surrounding environment, including glycoproteins, proteoglycans, and polysaccharides, which make the surface more wettable. The adhesion of these molecules provides nutrition and attachment points for organisms, affecting the adhesion and biofilm formation by microfouler organisms [21]. By a reversible process caused by different weak forces [22], as well as due to the bacterial organelles which promote cell attachment to the surfaces [23], the first cells adhere to the conditioning film surface. The irreversible adhesion of microfouler organisms and biofilm formation are driven by different types of physicochemical interactions with the surface, by the secretion of EPS from cells [21], and by quorum-sensing (QS) phenomena [24,25]. Biofilm development and maturation proceed with a greater production of EPS, which acts as a glue, having a significant impact on the cohesion and the protection of biofilms against environmental alterations and predation, as well as on the promotion of genetic information exchange [26,27]. Indeed, the EPS matrix may account for 50% to 90% of the biofilm composition, depending on the species present, the stage of biofilm development, and the environmental conditions [28]. The remaining percentage corresponds to the embedded organisms. The influence of biofilms on the settlement of macrofouling organisms is modulated by the spatial and temporal heterogeneity of marine environments, which suffer variations in terms of hydrodynamics, surface energy, topography, hydrophobicity, nutrients, and organic matter availability, as well as biological dispersal and aggregation at the microhabitat level [29,30]. Moreover, biological factors and ecological relationships such as parasitism, mutualism, commensalism, competition, and predation may affect macrofouling events (Figure 1).

**Figure 2:**
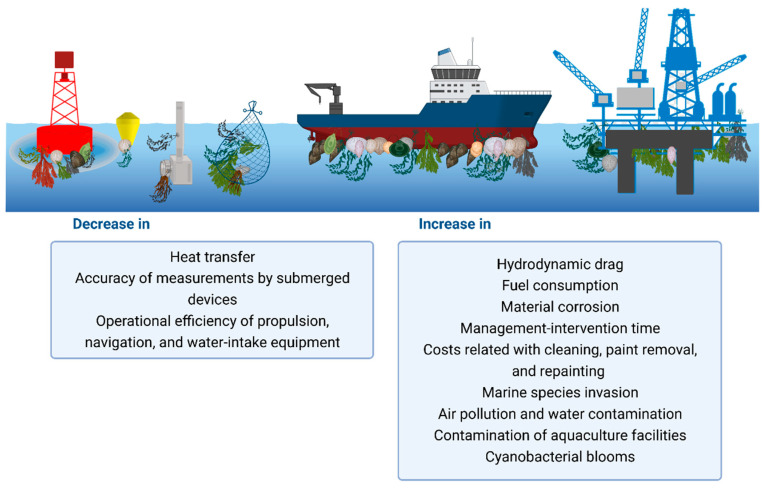
Main consequences of marine biofouling. This graphic representation shows the major effects of marine biofouling on submerged devices/equipment, such as sensors, buoys, cameras, aquaculture facilities, ships, and oil and gas platforms. This image was created with the software BioRender (https://biorender.com/).

The effects of marine biofouling involve an increase in direct costs either for maintenance or cleaning procedures, as well as indirect costs resulting from the efficiency loss of maritime industries. Additionally, issues related to human health, marine ecology, and the environment are also a matter of concern (Figure 2). The effect of marine biofouling on aquatic ecosystems is important as it disturbs species richness and genetic diversity [31]. Although several guidelines are discussed and implemented for the management of marine invasive species [5,18,32], the invasion of exotic species from different geographic areas continues to present a negative impact on global biodiversity since novel interactions between exotic and native species can be established, affecting predation and competition events [31,33]. Indirectly, marine biofouling contributes to climate change, environmental pollution, and global warming due to air pollution and greenhouse gas emissions promoted by the increased hydrodynamic drag and friction of vessels and ships [34]. Additional environmental and health-related problems involve the contamination of aquaculture facilities, such as fish cages and shellfish sites, the possibility of cyanobacterial blooms from benthic mat proliferations, and water contamination by the accumulation of toxins produced by some fouler organisms [6]. The economic impact of marine biofouling on industrial activities such as heat exchangers, water desalination stations, marine transport, aquaculture, gas, and oil industries remains relatively high. The direct economic costs of managing marine biofouling in the aquaculture industry are estimated to be around 10% of production costs [35]. The impacts on aquaculture infrastructures include the increased disease risk for marine animals, as well as human health effects due to biofoulers and associated pathogens, modified hydrodynamics in and around the cage affecting oxygen levels, water quality, and the cage’s volume and stability, increased weight, and physical damage that culminate in substantially reduced productivity [6]. In turn, in marine transport, around 35–50% of costs are concerned with increased fuel consumption [36], and in the gas and oil industry, about 20–30% are material corrosion costs [37]. In addition to the material corrosion of different facilities and infrastructures and costs related to cleaning, paint removal, and repainting, marine biofouling can prompt increased maintenance operations on submerged equipment. Moreover, specific areas of the vessels are highly prone to accumulating biofouling since they are often hidden, are difficult to inspect and treat, and can rapidly lose antifouling protection [38]. Examples of these niche areas include the internal pipework of vessels, dry-docking support strips, bow thrusters, rudders, and propeller shafts [39]. Additionally, a decrease in the precision of measurements on submerged devices, such as electrochemical and optical sensors, may also be promoted by the formation of a biofilm on the optics of these devices [40].

## 3. Marine Antifouling Strategies

**Figure 3:**
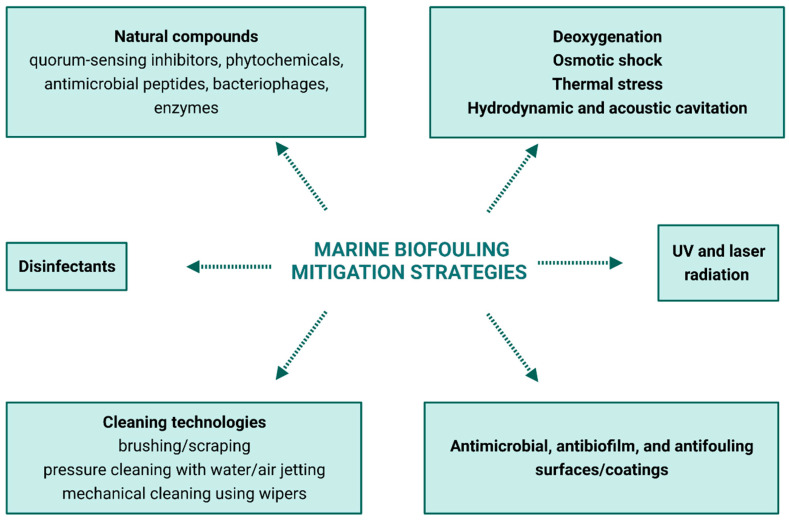
Preventive and control methodologies to mitigate marine biofouling effects.

Several strategies have been used to mitigate the effects of marine biofouling. These approaches can prevent and/or delay biofilm development and the attachment of macrofoulers, comprising antimicrobial, antibiofilm, and antifouling surfaces [41], or control already established biofilms and fouling communities (Figure 3, Table 1). Control methodologies involve using bacteriophages, enzymes, QS inhibitors, disinfectants, additional treatment methods, and cleaning technologies [38,42,43,44,45] (Figure 3). A range of criteria should be evaluated to select the most suitable marine antifouling strategy, including effectiveness, safety, biosecurity, compatibility with the materials of devices/equipment, and feasibility. First, effectiveness implies evaluating the activity, concentration, or intensity spectrum of antifouling activity and required exposure time. The antifouling strategy must be safe for the environment (ecotoxicological safety) and operators, as well as not exacerbate the biosecurity risk of releasing and establishing non-indigenous species. Moreover, the antifouling strategy should be compatible with the equipment itself to avoid damaging systems or other components of the devices/equipment. It should also be cost-effective and fulfill infrastructure requirements [38].

Antifouling paints containing arsenic, zinc, tin, and mercury were commonly used as the initial strategy to deal with marine biofouling [46,47] until their toxicity on the surrounding marine environment was demonstrated [48,49,50]. Indeed, in the 1960s, coatings incorporating a tributyl tin (TBT)-based biocide were the first to present robust effectiveness with a relatively low production cost. However, several findings indicated the negative impacts of TBT-based compounds related to their persistence and toxicity, showing adverse effects on non-target marine organisms. Several governments restricted its use, and the International Maritime Organization decided to ban the use of this type of biocide in the manufacturing of antifouling paints in 2003 and the presence of these paints on ship surfaces from 2008 [17].

Therefore, further biofouling treatments have been applied, including thermal stress, osmotic shock, deoxygenation, UV and laser radiation, and hydrodynamic and acoustic cavitation [38,44,45,51]. The most commonly available cleaning technologies are brushing, scraping, pressure cleaning with water/air jetting, or mechanical cleaning using wipers [33,38,44,45,51,52]. These mitigation strategies vary in their effectiveness in removing biofouling organisms and in their suitability for use on different marine surfaces. For instance, although the intensity of cavitation erosion of submerged surfaces depends on the material properties of the surface, liquid temperature, and the distance from the edge of the working tool to the fouling which should be removed, cavitation technology allows lower surface damage compared to brush-based technologies [53]. Moreover, nowadays, the cleaning of boats, ships, and additional moveable marine equipment such as cages and nets can be performed in a dry-dock or by in-water cleaning technologies [44,53]. Although in-water biofouling approaches can be cheaper than onshore activities, they may present higher chemical contamination and biosecurity risks, e.g., the application of underwater technology may increase the recolonization of surrounding surfaces [54].

Enzymes have also been proposed as an alternative to traditional antifouling compounds since they can act on the breakdown of adhesive components and the catalytic production of repellent compounds *in situ* [42]. A broad spectrum of aquatic disinfectants, such as Triple7 Enviroscale Plus^®^ (citric acid: 30–60%; lactic acid: 30–60%), Descalex^®^ (sulfamic acid: 60–100%), NALCO^®^ 79125 Safe Acid (sulfamic acid: 60–100%), and Rydlyme^®^ (hydrogen chloride: <10%), has been demonstrated to effectively control biofouling, being one of the most widespread treatments for cleaning and disinfecting marine equipment and devices [43,55,56]. They can be applied through the immersion of equipment into disinfectant solutions or spray applications since these disinfectants are available in powder and/or tablet form. TermoRens^®^ Liquid 104 cleansing fluid (5–15% citric acid and <10% phosphoric acid) was formulated to remove mussels, barnacles, and additional marine organisms and is marketed as environmentally friendly. Likewise, Barnacle Buster^®^ (85% phosphoric acid) is promoted as a safe, non-toxic, and biodegradable marine growth removal agent [38]. In the peroxygen family, Virkon^®^ Aquatic is 99.9% biodegradable and breaks down to water and oxygen [57]. It is one of the very few U.S. Environmental Protection Agency registered disinfectants labeled specifically for use in aquaculture facilities against aquatic bacterial, fungal, and viral pathogens, and is available through aquaculture suppliers such as Syndel in North America [58,59]. In turn, in the European Community, Antec International Limited indicates that the compound is registered as a disinfectant only for professional use. Due to the restrictive legislation, which requires several risk studies before registration and marketing authorization, the global costs of the development of new biocides or new antifouling coatings incorporating biocides have increased [17]. These costs reactivated the development of non-toxic approaches, including novel antifouling surfaces in which some natural compounds can be incorporated. Although the choice of the correct strategy depends on the cost and application possibilities, antifouling coatings are probably the most cost-effective method for boats and other submerged devices and equipment [60,61].

**Table 1:** Currently employed marine biofouling strategies, their advantages, and limitations.

Marine Biofouling Strategy	Description	Advantages	Limitations	Reference
Antimicrobial, antibiofilm, antifouling surfaces/coatings	Includes compounds (nanoparticles of copper, zinc, silver, immobilized molecules that become active upon contact, light-activated molecules) able to − kill or reduce the growth of foulers (antimicrobial) − decrease the ability to form and develop biofilms (antibiofilm) − reduce the adhesion/attachment of fouler organisms (antifouling)	− Probably represent the most cost-effective method against marine biofouling	− Coatings must be inert and transparent when applied to sensors requiring electrochemical or optical transduction	[45,61,62,63,64,65,66]
Natural compounds	Includes QS inhibitors, phytochemicals, peptides, bacteriophages, or specific enzymes which − degrade adhesives used for settlement − disrupt the biofilm matrix − interfere with intercellular communication	− Most of them can be incorporated on surfaces/coatings. − May be isolated from natural resources	− Compounds need to be produced in significant amounts	[42,67,68,69,70,71,72]
Disinfectants/chemical treatments	Mechanisms of action of disinfectants depend on the type/class but include the − damage and loss of the structural integrity of the cell wall and cytoplasmic membrane − leakage of intracellular components and cell lysis − inhibition of cellular metabolism/replication − denaturation of cellular constituents	− Compared to oxidizing treatment agents, non-oxidizing chemical treatment agents, such as quaternary ammonium compounds, can be more specific	− Insufficient information is available to accurately determine efficacy against all relevant biofouling taxa − Most of the chemical compound concentrations need to be actively monitored because their efficacy depends on different factors	[38,43,44,58,59]
Cleaning technologies	Commonly employed before other treatments and include physical removal by − brushing − scraping − pressure cleaning with water/air jetting − mechanical cleaning using wipers	− May be performed in a dry-dock or in water − Present fewer toxicological and environmental risks	− Associated with high maintenance costs and reduce the commercial operation time of ship hulls − Not entirely feasible when applied to sensors with sensitive components	[38,45]
UV and laser radiation	Radiation leads to the formation of toxic by-products	− A cheaper and more reliable application of UV radiation is likely to be a powerful approach − Requires low maintenance	− Incorporation into sensors has not been practical due to the high energy requirements − Can be better suited as a pretreatment rather than a final strategy against marine biofouling − Difficult to apply to large, submerged structures	[44,45]
Thermal stress	Heating seawater to above the thermal tolerance of biofouling organisms	− Well-suited for application to internal pipework, given the confined spaces and relatively small total volumes to be treated − Resilient taxa can render it nonviable in 2 h or less − It poses few risks to operators and is unlikely to harm vessel components at or below 60 °C − Fewer toxicological and environmental risks are presented	− It is only fitted to confined spaces − It requires continual monitoring of water temperature to ensure lethal conditions are maintained throughout the process	[38]
Deoxygenation	Reducing dissolved oxygen concentrations to below the tolerance of biofouling organisms by wrapping fouled surfaces with impermeable plastic	− It enables vessels to be treated *in situ*, preventing the expense of removing boats from the water − Fewer toxicological and environmental risks	− It can take several weeks to kill resilient fouling taxa − Absolute anoxic conditions may be required to expose all taxa to lethal conditions	[38,73,74,75]
Hydrodynamic cavitation Acoustic cavitation (by ultrasonic irradiation)	Hydrodynamic mode—cavitation is produced by pressure variations obtained using the geometry of the system, creating velocity variation Acoustic cavitation—the pressure variations in the liquid are accomplished using sound waves, usually high-intensity ultrasound (16 kHz–1 MHz), which creates high liquid shear forces that prevent the settlement of organisms on the submerged surfaces	− They seem to have no adverse effects on marine life	− It may be limited by energy costs − The installation of ultrasonic treatment systems is expensive − Further research is required to optimize operating parameters accounting for the effects of acoustic treatments on coating integrity and the influence of pressure waves on the viscoelastic properties of biofilms	[44,45,76,77]
Osmotic shock	Reducing salinity interferes with the osmotic balance of marine organisms	− Fewer toxicological and environmental risks associated	− It is unlikely to be effective within acceptable timeframes − Some marine bivalves can survive weeks in freshwater	[38]

### 3.1. Marine Coatings

Among all the strategies presented, novel modified surfaces and coatings probably represent the most cost-effective and promising methodology to tackle marine biofouling. These approaches include preventive measures for adhesion, biofilm formation, and development, and consequently delay macrofouler attachment and settlement. Since microfouling events can be managed directly by the performance of these surfaces/coatings, the effects of macrofoulers can be controlled more effectively. Antifouling coatings can be divided into chemically bioactive coatings and biocide-free coatings. The chemically active antifouling technologies, which act through the controlled release of bioactive molecules (most recently booster biocides), can be subdivided into three main categories: contact-leaching coatings, controlled-depletion paints (CDPs), and self-polishing copolymers (SPCs), and a few combinations thereof. All these technologies control the release of bioactive molecules via various chemical mechanisms, many of which remain partially understood [78]. From those, SPC coatings are the most successful antifouling coating technology in terms of long-term efficiency in service life, and where the biocidal compound is chemically bonded to the binder, which is gradually hydrolyzed and dissolved in water to release the antifouling bioactive agent. On the other hand, among the biocide-free coatings technologies, fouling-release coatings (FRCs) are the most acceptable and implemented in the marine industry, mostly allied to their eco-friendly biocide-free antifouling effect, acting through mechanical and physicochemical mechanisms and providing long-term efficiency, particularly for dynamic systems (e.g., ships) [79].

Although the first SPC included TBT [47], novel coatings have been developed. In turn, with FRCs, biofouling may be removed by hydrodynamic stress through ship movement or mechanical cleaning. Although they prevent macrofouling events under dynamic conditions, FRCs are less effective in preventing the formation of the first adhesion layers [79].

In recent years, bioinspired antifouling strategies have emerged, including micro- and nanostructured surfaces, natural bioactive compounds, bioinspired hydrogels, slippery liquid-infused porous surfaces, bioinspired dynamic surfaces, and zwitterionic/amphoteric coatings [63,64]. Due to natural evolution, different organisms, including mussels, crabs, sharks, and insects, have demonstrated natural antifouling abilities in their bodies and structures [61]. Bioinspired coatings aim to mimic shapes, functions, and elements of nature. Since these promising antifouling coatings show practical value due to their environmental compatibility, they have been intensively explored to deal with marine biofouling. Biomimetic surfaces may be produced by several techniques, including deposition and electrostatic methods, 3D printing, self-assembly, and lithography, the most common methodology [63]. Most biomimetic coatings have been produced from soft polymers, such as polydimethylsiloxane (PDMS), poly(methyl methacrylate (PMMA), silicone, polyurethane, and polypropylene, since they present a low elastic modulus (a measure of a material’s stiffness or resistance to elastic deformation under stress, calculated by the ratio of stress and strain, corresponding to the stress of the material) and low surface energy, allowing a fouling release effect [63]. Moreover, they are also inexpensive and chemically inert. Indeed, a surface based on shark skin comprising microscopic features (Sharklet AF^TM^) was developed to prevent bacterial adhesion and biofilm development [80]. The drawbacks of biomimetic surfaces include the possibility of the designed nano- or microstructure being only active against specific fouling organisms, thus limiting the application range. Moreover, the antifouling effect may decrease after some time due to fouling organisms’ attachment [61]. In addition, a low-cost and simple fabrication approach is required for marine applications [61].

Natural antifouling compounds obtained from invertebrates, plants, and microorganisms have also been proposed as one of the best alternatives to current chemical formulations in marine paints and coatings [66,67,68,69,70,81]. Antifouling mechanisms of these compounds may be related to alterations in protein expression (e.g., by promoting the underexpression of proteins related to adhesion and biofilm development), oxidative stress induction, neurotransmission blocking (caused by, for example, the inhibition of acetylcholine esterase activity, which interrupts cholinergic signaling and reduces the success of the settlement of fouling organisms), surface modification (e.g., by blocking the attachment site of bacteria), and biofilm inhibition through different mechanisms. However, the molecular mechanisms of action of these compounds are still under analysis. Compared to natural compounds obtained from higher organisms, such as crustacean shells and mollusks [82], those sourced from microorganisms present several benefits since they may be produced at a low cost by optimizing cultivation conditions [61,69]. Some of them are isolated from marine microorganisms [83], such as chitosan and melanin [84,85]. For example, antimicrobial peptides, commonly classified according to their source, charge, structure or residual pattern, and function (antibacterial, antibiofilm, antifungal, antiparasitic, insecticidal), include both membrane-acting and non-membrane-acting peptides [70]. The advantages of marine antimicrobial peptides include their stability in high salt concentrations and a range of temperatures (4 °C to 20 °C) [86]. Additionally, the use of extracts instead of purified compounds previously identified as active molecules could be a suitable approach due to lower production costs and the possibility of having different bioactive compounds in the same extract that may act synergically on different targets of fouler organisms [71,81].

Whales, fishes, and amphibians also secrete specific mucus that can prevent microbial adhesion, known as natural hydrogels [61,63]. Researchers have prepared synthetic hydrogels with a high degree of similarity to these natural hydrogels. Hydrogels are particularly hydrophilic 3D network structures of soft material that can absorb water, exhibiting a low interfacial free energy when in contact with liquid and a good resistance to protein adsorption [61,63]. Once a hydrogen bonding or an electrostatically induced hydration layer is formed on a hydrophilic surface, this constitutes a physical barrier to the adhesion and attachment of fouling organisms [87]. Synthetic hydrogels, such as polyethylene glycol (PEG), polyacrylamide (PAM), and polyurethane (PU), are usually fabricated by physical and chemical cross-linking methods [88]. However, improving the mechanical strength of hydrogels is required for their application in harsh marine environments. Filling hydrogels/polymers with nanomaterials and their modification with polymer brushes is an effective antifouling strategy since the brushes act as a steric barrier for bacteria and large molecules [14,63,89,90]. Recently, corals have been a subject of great interest for researchers as a novel source for exploring the potential of biomimetic surfaces [66]. Antifouling strategies from these organisms are related to the production of natural antifouling substances but are also due to their foul release, sloughing, and fluorescence effect. Indeed, the mucus produced by corals can protect them from biofouling by presenting a physical barrier, the production of antimicrobial compounds, and a slime sloughing effect. Furthermore, fluorescent corals emit a weak light that may prevent the attachment of diatoms. As no fluorescence effect was observed on bacteria, this strategy must be combined with additional ones to attain a broad-spectrum antifouling capability. However, the main drawbacks of corals are related to their natural environments. Since coral reefs are ecologically sensitive, their use may damage their ecosystems [66]. Although natural antifouling substances have been isolated from marine microbial organisms, invertebrates, algae, corals, and plants, chemical synthesis based on their composition is an alternative approach to tackle their limited production and extraction, which may hinder their large-scale production [61].

Slippery liquid-infused porous surfaces consist of a porous/textured material and lubricating liquid [63,64]. The advantages of these surfaces include the repellence of different liquids and resistance to ice and high pressures. These act as a physical barrier and a molecularly smooth surface, decreasing attachment strength and blocking signals with self-cleaning properties [63]. However, due to the complexity of the marine environment, the stability of these surfaces remains a challenge since the lubricant is easily lost under shear flow [64]. Dynamic surfaces, a changing surface that renews itself in seawater while removing fouling organisms, are an additional bioinspired strategy [63] using self-polishing and degradable copolymers. Finally, zwitterionic/amphoteric coatings are also a promising bioinspired approach [61,63,91]. The constituent of the lipid outer layer of the cell membrane, phosphatidylcholine, is an amphiphilic molecule comprising a hydrophilic head and a hydrophobic tail, showing great resistance to protein binding [61]. The phosphatidylcholine head groups are zwitterions consisting of equal numbers of oppositely charged species exhibiting neutral charge and a hydrophilic character. Zwitterionic polymers have the same number of cations and anions along their polymer chains [63], forming a strong hydration layer that impacts the initial deposition of proteins, contributing to their antifouling ability [14]. The main advantage of using zwitterionic polymer brushes in marine environments is that they are not affected by high concentrations of salt ions [63]. In turn, poor antifouling durability and mechanical strength are some of their limitations.

Polymer brushes are polymeric assemblies tethered at one end to a solid substrate either through covalent attachment or physical adsorption [92]. Antifouling polymer brushes have been developed to prevent the adsorption of molecules and adhesion by limiting the contact of the surface with the organism and reducing the force involved in bacterial attachment [93]. The immobilization of antimicrobial peptides, which present a broad spectrum of activity, on polymer brushes also represents a good approach to creating surfaces with antibacterial properties [70].

According to surface wettability, antifouling coatings may be considered hydrophilic, hydrophobic, or amphiphilic coatings. Hydrophilic coatings, such as hydrogels, form a hydrated layer that may bind water molecules so strongly that other molecules and fouling organisms cannot replace them during adhesion, thus preventing initial biofouling. However, their antifouling performance is not long-lasting, and their mechanical strength is usually low [64]. Hydrophobic coatings, such as PDMS, exhibit low surface energies, reducing the adhesion strength of fouling organisms on surfaces and allowing their easy removal [62]. However, due to the hydrophobic interaction between the slime compositions and hydrophobic surfaces, they cannot prevent the development of the first slime layer of the biofilm, which is mainly composed of proteins, bacteria, and diatoms [64]. Therefore, amphiphilic coatings, which are characterized by the presence of hydrophilic and hydrophobic groups, combine the advantages of hydrophilic and hydrophobic surfaces. Moreover, superhydrophobic surfaces, which present a high water contact angle, typically > 150°, have also been tested as improvements for marine surfaces [94]. Studies performed by Ellinas et al. [95] and Kefallinou et al. [16] described hybrid metal-sputtered superhydrophobic surfaces demonstrating both bacterial repulsion and long-term killing efficacy against the cyanobacteria *Synechococcus* sp. PCC7942. Among the two low-surface-energy hydrophobic coatings used, a chlorosilane and a fluorocarbon coating, the fluorocarbon layer managed to better maintain the superhydrophobicity and anti-adhesiveness of the surface when enriched with an adequate amount of copper [16]. These bifunctional surfaces with antifouling and bactericidal activity can be a promising strategy for managing marine biofouling. Although several methods have been developed to produce superhydrophobic surfaces, including layer-by-layer assembly, electrodeposition, photolithography, electrospinning, and 3D printing, those based on simple chemical reactions are more attractive due to their simple procedure, low cost, and large-scale production potential [64].

Nanotechnology-based coatings, including the use of silver nanoparticles [96,97,98], carbon nanotubes (CNTs) [99,100,101,102], graphene [103,104,105,106,107], and metal oxide semiconductors such as titanium dioxide (TiO_2_) [108] and zinc oxide (ZnO) [79], are relevant novel approaches to prevent biofouling. Photocatalytic antifouling coatings, based on the redox ability of semiconductor photocatalysts under light conditions such as TiO_2_ and ZnO, showed good chemical stability and antifouling performance under ultraviolet conditions [62]. For example, ZnO can produce reactive oxygen species that induce oxidative stress and intracellular component outflow [64]. Marine coatings containing nanomaterials have been reported to be an efficient antifouling strategy offering hydrophobicity, water repellency, high durability, and anti-corrosive properties [65]. Moreover, nanocomposite coatings have good adhesion between the coating and the hull. Using a nanocomposite coating on the metal surface of a hull may eliminate the presence of holes, which can contribute to corrosion [64].

Although the main concern in marine coatings is related to their antifouling performance, the corrosion performance should also be considered [64]. Indeed, the adhesion and attachment of organisms on surfaces increase changes in the concentrations of ions, oxygen levels, redox potential, conductivity, and pH, which in turn prompt the biodegradation of coatings and stimulate chemical and electrochemical reactions between organisms, media, and metals. This type of corrosion, the microbially influenced corrosion, is responsible for about 20% of the corrosion occurring in aqueous environments. Amphiphilic polymers, bioinspired superhydrophobic surfaces, and slippery liquid-infused porous surfaces represent inherently integrated antifouling and anticorrosion coatings [64]. However, some modifications, such as the integration of polydopamine, graphene, polyaniline, and amorphous carbon, can be performed on other types of coatings to enhance their anticorrosion properties [64].

Due to the wide distribution of marine environments, a key question remains: how can the scientific community transfer *in vitro* knowledge from the laboratory to natural marine environments?

#### 3.1.1. *In Vitro* Studies

Although numerous *in vitro* systems, including microtiter plates, the Calgary device, flow chambers and flow cells, the Robbins device, rotary biofilm reactors, and microfluidic devices, have been designed to study biofilm formation to better mimic real development conditions [12,13], few of them are characterized to be used in marine biofouling studies.

*In vitro* studies are very important since they are necessary as the first approach to evaluate the effectiveness of specific marine coatings on biofilms and to collect useful information for further *in situ* studies (Table 2). *In vitro* models can be operated in static or dynamic conditions. Although static models are simple and cheap and do not require specialized equipment, they often do not accurately represent many environmental conditions (e.g., the hydrodynamics) of natural marine environments [109]. In a dynamic model, nutrient supply and metabolite removal often occur throughout the process, resulting in longer-lasting operation. They also take into account the presence of hydrodynamic conditions, which have a real impact on biofilm development [110]. However, they often require specific equipment, higher costs, and technical competency due to their complexity of use [111].

*In vitro* models provide well-defined results, allowing precise control of experimental parameters while concomitantly allowing single variables to change. Consequently, this allows the study of the effects of single elements on various aspects of biofilm development. This simplistic approach is not possible for *in situ* models due to natural variations. However, they lack the interaction between different marine organisms since some species act synergistically through metabolite and signal production and/or direct contact, as well as all changes associated with environmental parameters that occur in real aquatic environments, such as pH, temperature, and hydrodynamic condition variations. Moreover, many *in vitro* studies are performed using non-representative artificial media [94,112] and hydrodynamic conditions [60,97]. This is particularly important since in marine environments, surfaces are in contact with a wide range of hydrodynamic conditions according to their location, and fouling organisms have evolved their ability to settle and proliferate on a range of surfaces, either under lower hydrodynamic conditions, such as a shear rate of 50 s^−1^, reported for a ship in a harbor, and under high turbulent conditions, such as at 125,000 s^−1^, the reported shear rate value for a navigating ship [113,114]. Likewise, hydrodynamic conditions also play a pivotal role in biofilm development since they affect biofilm architecture, diversity, EPS production, energy metabolism, and mass transfer, prompting molecular changes [30,115,116,117]. While higher flow velocities improve molecular transport by convection, the higher density of biofilms decreases the diffusivity of the molecules inside them [118]. Furthermore, stronger shear forces are responsible for higher biofilm sloughing or detachment [119]. Indeed, to study how microalgae biofilms respond to different hydrodynamic conditions, the architecture and cohesion of *Chlorella vulgaris* biofilms were investigated in flow cells at three different shear stresses: 1, 6.5, and 11 mPa [2]. Biofilm cohesion was heterogeneous at low shear stress, resulting in a strong layer close to the substrate and more loose superficial ones. In turn, higher shear stress increased the cohesion of the biofilms allowing them to grow thicker and produce more biomass [2]. Using a microfluidic flow cell, the impact of shear stress on *Cobetia marina* and *Pseudomonas aeruginosa* biofilm formation was also evaluated [3]. The results indicated that hydrodynamics affect the biomass, maximum thickness, and surface area of biofilms, with the higher shear stress (5.6 Pa) promoting thinner biofilms than the lower shear stress (0.2 Pa). Particularly on cyanobacterial biofilms, studies performed on coccoid [120,121] and filamentous [122,123,124,125] cyanobacteria at controlled hydrodynamic conditions (values of shear rate of 4 s^−1^ and 40 s^−1^) showed a higher biofilm development at the lower shear rate. A study that aimed to evaluate the settlement of diatoms on different antifouling coatings also revealed that biofilm adhesion, diatom abundance, and diversity were found to be significantly different between static and dynamic treatments [126]. Therefore, due to the importance of hydrodynamic conditions on biofilm development, *in vitro* studies which aim to evaluate the performance of novel antifouling coatings [90,127] should mimic the typical real conditions that prevail in marine environments to bring the *in vitro* operational conditions closer to the natural aquatic environments [122]. Because of reactor geometry on the flow, the shear stress or shear rate should be considered to characterize shear effects/hydrodynamic conditions. The shear rate is the derivative of the velocity in the perpendicular direction from the wall system [128], quantifying the frequency at which cells contact the surface. The shear stress in Newtonian fluids is proportional to the shear rate, where the fluid viscosity is the constant of proportionality [128], representing the friction from the fluid acting on the adhered cells/biofilm. Computational fluid dynamics (CFD) is a commonly used approach to model biofilm reactors since it enables the faster estimation of the fluid flow parameters of these systems and at a relatively low cost in comparison to experimental techniques [129]. Since the results obtained from CFD comprise a larger number of points in the flow path, they provide much more detailed information about the flow field when compared with the experimental approach [113], although validation of the simulation results is required. Moreover, the standardization of biofilm reactors and operation conditions enables a rigorous comparison of hydrodynamic data obtained from different laboratories.

Microfluidic devices have demonstrated high potential and versatility for the study of biofilm formation under different growth conditions. These platforms allow the testing of different materials at highly controlled hydrodynamic conditions through a precise, non-invasive, and real-time analysis [130]. A microfluidic assay used to quantify how easily diatoms can be removed from several surfaces through shear force application showed that, while the number of adhered cells was barely affected by the different coatings, the critical shear stress required for their removal varied significantly [131]. Although these devices require small volumes to operate and can be custom-made for specific purposes, they require special equipment for manufacturing and operation. Moreover, clogging events can occur due to the small dimensions, air bubbles can have a very significant effect, and viscosity effects are also critical [13]. In addition to microfluidic devices [3,131,132,133], platforms that specifically evaluate macrofouling development [134,135,136] have also been used in marine biofouling studies. Recently, a CFD analysis performed on agitated 12-well microtiter plates showed that this platform can be used as a marine biofilm reactor to mimic marine environments since the shear rate range achieved comprises the values found in real aquatic environments [122]. Indeed, the use of agitated microtiter plates at defined hydrodynamic conditions can be a very suitable and reasonable approach since this platform requires low volumes (and consequently has reduced costs) and is easy to handle. Furthermore, it enables the control of different parameters and the use of coupons from several materials to test the impact of different surfaces and coatings in marine biofouling, in a high-throughput mode (often required for studies performed during long time intervals with sampling on different days).

*In vitro* models still also face an important drawback related to the use of unrepresentative fouler organisms. Indeed, some of these studies, which aim to evaluate novel coatings to tackle marine biofouling, are performed using common model organisms for biofilm studies, such as *Escherichia coli*, *Staphylococcus aureus*, *Pseudomonas aeruginosa*, *Bacillus* sp., and *Candida albicans*, but they are not considered relevant microfoulers in marine environments [4,107,137,138]. These models often use a single bacterial species, which is never the case in a natural environment since most biofilm communities are composed of multiple organisms living in proximity. Therefore, organism diversity should be considered in the evaluation of the performance of novel antifouling surfaces, as well as it is important to consider organisms with strong fouling activity and wide global distribution. However, some of the barriers to the *in vitro* study of mixed biofilms are related to the lack of knowledge about the abundance of each biofilm resident, which makes it difficult to select the correct initial concentration for *in vitro* assays, i.e., the difficulty in labeling different populations on biofilms (common issues are the stability of the tag and the influence it may have on the microorganism physiology) and the overall challenge in interpreting inter-species relations [1,139]. The relevance of using mixed populations instead of single cultures for *in vitro* screening assays of marine antifouling coatings was assessed in a recent study in which single- and dual-species biofilms of *Pseudoalteromonas tunicata* and a coccoid cyanobacterium were grown for 49 days on an epoxy resin [139], a marine coating with known antifouling potential [120]. The results obtained suggest that for initial screening, starting with a single representative organism such as a cyanobacterium is a good approach to predict the results obtainedin marine environments by *in vitro* testing. Indeed, while a marine bacterium alone revealed biofilm growth kinetics similar to dual-species biofilms, single-species biofilms presented a higher number of cells, biofilm wet weight, thickness, and biovolume when compared to dual-species biofilms [139]. Therefore, in that particular case, single-species cyanobacterial biofilms corresponded to the worst-case scenario for testing.

Although models that study early stages of biofouling formation are easier to implement due to the heterogeneity, complexity, and evolving nature of marine biofilms, the design of an accurate marine *in vitro* model is extremely challenging. To improve the evaluation of the performance of novel marine coatings, the characterization of marine reactors and the operational conditions that enable them to mimic, as closely as possible, the marine environment should be considered.

**Table 2:** *In vitro* studies focused on different surfaces/coatings used in marine environments. The different surfaces/coatings were divided into non-modified surfaces, chemically bioactive coatings, biocide-free coatings, and a combined strategy of chemically bioactive coatings and biocide-free coatings. The distribution by rows follows a chronological order.

**Non-Modified Surfaces**				
**Surface/Coatings**	**Organism**	**Experimental Setup**	**Major Findings**	**Reference**
Glass Perspex	*Leptolyngbya mycoidea* LEGE 06118 (filamentous cyanobacterium)	Dynamic assay (shear rate 40 s^−1^) Z8 medium 3 weeks, 25 °C	− Higher biofilm development on Perspex	[140]
Polyethylene Polypropylene Polyethylene terephthalate PVC	*Escherichia coli* *Bacillus subtilis* *Bacillus pumilus* (bacteria)	Static assay Short-term attachment: 10 min Long-term attachment: 4 h to 16 h 30/37 °C	− Higher adhesion to polyethylene and PVC compared to polypropylene and polyethylene terephthalate − Surface hardness modulated bacterial adhesion	[4]
Glass Perspex	*Nodosilinea* sp. LEGE 06020 *Nodosilinea* sp. LEGE 06022 Unidentified filamentous Synechococcales LEGE 07185 (filamentous cyanobacteria)	Dynamic assay (shear rate 4 and 40 s^−1^) Z8 medium 7 weeks, 25 °C	− *Nodosilinea* sp. LEGE 06022 developed a higher amount of biofilm on Perspex	[122]
	*Nodosilinea* sp. LEGE 06145 *Nodosilinea* sp. LEGE 0611 (filamentous cyanobacteria)		− Higher biofilm formation on glass at 4 s^−1^, and on Perspex at 40 s^−1^ − Surfaces affect biofilm protein composition	[123]
PMMA Glass	*Phormidium* AP3 *Phormidium* AP9F (filamentous cyanobacteria)	ASN-III medium 30 days	− Higher biofilm development was observed in PMMA than in glass flasks	[141]
Glass Perspex	Unidentified filamentous cyanobacterium LEGE 06007 (filamentous cyanobacterium)	Dynamic assay (shear rate 4 and 40 s^−1^) Z8 medium 7 weeks, 25 °C	− Differentially expressed proteins between surfaces included a beta-propeller domain-containing protein, chaperone DnaK, SLH-domain-containing proteins, an OMF-family outer-membrane protein, and uncharacterized proteins	[124]
	*Leptothoe* sp. LEGE 181153 *Jaaginema* sp. LEGE 191154 (filamentous cyanobacteria)	Dynamic assay (shear rate 40 s^−1^) Z8 medium 7 weeks, 25 °C	− CLSM analysis showed different patterns between both cyanobacterial strains and also among different surfaces	[142]
**Chemically bioactive coatings**				
**Surface/coatings**	**Organism**	**Experimental setup**	**Major findings**	**Reference**
Graphene oxide in alkyd resin surface	*Escherichia coli* *Staphylococcus aureus* *Pseudomonas aeruginosa* (bacteria)	Static assayNutrient medium24 and 48 h Room temperature	− Graphene-oxide-coated surfaces reduced bacterial growth (up to 94% loss of cell viability) and long-time exposure increased the death rate − Good corrosion-resistance behavior was observed	[137]
Graphene-coated silica	*Halomonas* spp. (bacterium)	Static assay Saline solution (0.5 wt%), 72 h, 20 °C	− Expression levels of adhesion genes were reduced − No bactericide effect of graphene coatings was observed	[60]
Cationic polymer brush (PDMAEMA) Anionic polymer brush (PSPMA) Neutral polymer brush (PHEMA-co-PEG10MA) Zwitterionic polymer brush (PSBMA)	*Cobetia marina* (bacterium) *Ulva linza* (green alga) *Balanus amphitrite Balanus improvises* (barnacles)	Static/dynamic assays Tropic Marin artificial seawater Attachment of *Cobetia marina* (50 rpm, 1 h, room temperature) Settlement (static, 45 min) Adhesion strength of *Ulva linza* zoospores (shear stress of 52 Pa, 5 min) Settlement of barnacles (24 h and 48 h, 28 °C)	− PSPMA showed good resistance toward attachment of *Cobetia marina* and *Ulva linza* zoospores − Lower settlement of barnacles on zwitterionic PSBMA and on a neutral polymer brush	[89]
Glass PDMS Multi-walled carbon nanotube (MWCNT)–PDMS surfaces Titanium dioxide–PDMS surfaces	*Mytilus coruscus* (mussel)	Static assayAutoclaved filtered seawater, 12 h, 18 °C	− Incorporation of CNTs and titanium dioxide in PDMS inhibited the settlement of mussels	[108]
Graphene–silver nanocomposites	*Halomonas pacifica* (bacterium) *Dunaliella tertiolecta* *Isochrysis* sp. (microalgae)	Static assayMarine broth, 24 h, 26 °C Static assay Provasoli medium 4 days	− Nanocomposite inhibited biofilm formation (99.6% reduction) and had antiproliferative effects on marine microalgae (growth inhibition greater than 80%)	[97]
Graphene oxide–alumina nanorod–PDMS nanocomposites	*Micrococcus* sp. *Pseudomonas putida* (bacteria) *Aspergillus niger* (fungus)	Nutrient-infused medium28 days, 35 °C	− Nanocomposite showed high adhesion resistance (approximately 95% reduction)	[94]
Hydroxyl-modified MWCNT–silicone-oil-infused PDMS coatings	Marine bacteria	Fresh seawater10 days, 28 °C	− Anti-adhesion and antifouling properties were enhanced when higher volume ratios of hydroxylated MWCNTs were used	[102]
Graphene oxide–polymeric membrane calcium-ion-selective electrode sensor	Marine bacteria	Luria–Bertani medium1 h and 5 h Room temperature	− Graphene-oxide-coated sensor inhibited biofilm formation	[112]
Graphene oxide-–silver nanoparticle–PDMS–silica coatings	*Escherichia coli* (bacterium) *Phaeodactylum tricornutum* *Navicula torguatum* (diatoms) *Chlorella* sp. (algae)	Bacterial test under dynamic conditions Saline solution (0.9 wt%), 24 h, 37 °C Antialgae test 24 h	− Coating containing silver nanoparticles showed antibacterial and antialgal (up to 17% reduction in surface coverage) properties	[96]
Graphene oxide-–silica nanoparticle–PDMS composite coatings on carbon steel surfaces	*Pseudomonas* sp. *Bacillus* sp. (bacteria) Freshwater bacterial culture	Nutrient broth 72 h	− Efficiency of the coated surfaces was 99.9% against *Bacillus* sp. in freshwater culture and 89.6% against *Pseudomonas* sp.	[143]
Epoxy-matrix polyaniline/*p*-phenylenediamine-functionalized graphene oxide coatings	Organisms in simulated marine environment (including guppy fish, algae, and dwarf hair grass)	Static assay 3 months, 25–27 °C	− Anticorrosion and antifouling properties were enhanced in the functionalized graphene oxide composite	[105]
Laser-induced graphene coatings	*Cobetia marina* (bacterium)	Dynamic assay (65 rpm)Artificial seawater1 and 36 h	− Laser-induced graphene coatings showed greater initial bacterial attachment but up to 80% less bacterial coverage after 36 h − Initial attachment rates were reduced by the application of negative or positive potential	[144]
Methanol cell extract (MCE) from *Bacillus licheniformis*	*Vibrio aestuarianus Vibrio tubiashii* *Pseudoalteromonas flavipulchra* *Pseudoalteromonas* *maricaloris* (bacteria) *Bugula neritina* (bryozoan) *Amphibalanus amphitrite* (barnacle) *Artemia salina* (marine invertebrate)	Static assays Bacterial biofilm assay Tryptone Soya broth 22 °C, 44 h *Bugula neritina* settlement assay Filtered seawater 20 °C 24 and 48 h *Amphibalanus amphitrite* settlement assay Filtered seawater 28 °C 24 and 48 h *Artemia salina* toxicity assay Filtered seawater 25 °C, 24 h	− MCE inhibited bacterial biofilm formation and displayed considerable efficacy in preventing the settlement of *Bugula neritina* without inducing lethality − MCE presented low toxicity against the non-target *Artemia salina*	[81]
Pristine silicon rubber Graphene-added silicon rubber Graphene-added silicon rubber filled with quaternary ammonium salt coatings	*Paracoccus pantotrophus* (bacterium) *Chlorella* *pyrenoidosa*(alga)	Static conditions 24 and 48 h 37 °C (bacterium) 2 and 4 days, 25 °C (algae)	− Bactericidal graphene-added silicon rubber filled with quaternary ammonium salt coating showed an anti-adhesion effect	[106]
Guanidine-functionalized graphene/boron acrylate polymer composite	*Escherichia coli* *Staphylococcus aureus* (bacteria) *Phaeodactylum tricornutumNitzschia closterium f. minutíssimaHalamphora* sp. (diatoms)	Luria–Bertani medium12 h, 37 °CF/2 medium14 days, 21 °C	− Coatings presented excellent antibacterial properties (up to 94.2% and 95% reduction for *E. coli* and *S. aureus*, respectively) and diatom anti-adhesion (up to 99.2%)	[145]
Poly(lactic acid) (PLA)–chitosan (CS) surfaces	*Cobetia marina* (bacterium)	Dynamic assay (shear rate 40 s^−1^) Väätänen Nine-Salt Solution medium 7 weeks, 25 °C	− PLA–CS surfaces were able to reduce the number of culturable cells by up to 68% and biofilm thickness by up to 36%	[84]
GBA26 *** (synthetic gallic acid derivative) incorporated in a polyurethane-based coating	*Pseudoalteromonas tunicate* (bacterium)	Dynamic assay (shear rate 40 s^−1^) Väätänen Nine-Salt Solution medium 7 weeks, 25 °C	− Polyurethane-based coating containing 2 wt% GBA26 and the trimethylolpropane triaziridine propionate cross-linker provided the best long-term performance	[146]
Epoxy-coated glass surface containing 5 wt% GNP	*Lusitaniella coriacea* LEGE 07157 (filamentous cyanobacterium)	Dynamic assay (shear rate 40 s^−1^) Z8 medium 7 weeks, 25 °C	− Biofilms formed on composite presented a 44% reduction in biofilm wet weight, 54% in biofilm thickness, 82% in biovolume, and 64% in surface coverage compared to epoxy-coated glass	[103]
Epoxy-coated glass surface containing 3 wt% CNT	*Nodosilinea* cf. *nodulosa* LEGE 10377 (filamentous cyanobacterium)	Dynamic assay (shear rate 40 s^−1^) Z8 medium 7 weeks, 25 °C	− A decrease in biofilm wet weight, thickness, and biovolume was reached with the CNT composite	[99]
PDMS surface containing 5 wt% GNP	*Cobetia marina* (bacterium)	Dynamic assay (shear rate 40 s^−1^) Väätänen Nine-Salt Solution medium 6 weeks, 25 °C	− Biofilm formation was reduced on the composite (lower total cell number and up to 43% thickness reduction)	[147]
Chitosan–melanin hybrid nanoparticle coatings	*E. coli* *S. aureus(bacteria)* *Isochrysis galbana* *(microalga)* *Artemia salina* (marine invertebrate) *Amphibalanus amphitrite* (barnacle)	MIC of bacterial strains 37 °C, 120 rpm, 24 h and 48 h Antialgal activity 25 °C, 12:12 light–dark cycle, 48 h Cytotoxicity assay 24 h and 48 h	− Chitosan–melanin hybrid nanoparticle had antibacterial activity against *E. coli* and *S. aureus* (MIC of 1.56 μg/mL and 0.871 μg/mL, respectively) and antialgal activity against *Isochrysis galbana* (IC50 value after 48 h was 0.176 mg/mL) − Low toxicity to *A. salina* and *A. amphitrite* nauplii (after 24 h, LC50 values of exposed *A. salina* and *A. amphitrite* nauplii were 397 and 250 mg/mL)	[85]
**Biocide-free coatings**				
**Surface/coatings**	**Organism**	**Experimental setup**	**Major findings**	**Reference**
Epoxy-coated glass Silicone hydrogel coating	*Cyanobium* sp. LEGE 10375 (unicellular cyanobacterium) *Pseudoalteromonas tunicata* (bacterium)	Dynamic assay (shear rate 40 s^−1^) Z8 medium 7 weeks, 25 °C	− Epoxy-coated glass surfaces were effective in inhibiting biofilm formation at the initial stages, while the silicone hydrogel coating showed high antibiofilm efficacy during biofilm maturation − Silicone hydrogel was less prone to biofilm formation, and its efficacy may be dependent on the fouling microorganism	[148]
Glass Epoxy-coated glass	*Synechocystis salina* LEGE 00041 *Cyanobium sp.* LEGE 06097 (unicellular cyanobacteria)	Dynamic assay (shear rate 40 s^−1^) Z8 medium 24 h and 6 weeks 25° C	− Antibiofilm performance of the epoxy-coated glass was observed	[120]
Glass Perspex Polystyrene Epoxy-coated glass Silicone hydrogel coating	*Synechocystis salina* LEGE 00041 *Cyanobium* sp. LEGE 06098 *Cyanobium* sp. LEGE 10375 (unicellular cyanobacteria)	Dynamic assay (shear rate 40 s^−1^) Z8 medium 7 weeks, 25 °C	− Silicone hydrogel coating was effective in inhibiting biofilm formation. − Cyanobacterial biofilms formed on silicone hydrogel coating showed a lower percentage and size of empty spaces among all tested surfaces.	[149]
Glass Epoxy-coated glass	*Synechocystis salina* LEGE 00041 *Synechocystis salina* LEGE 06155 *Cyanobium* sp. LEGE 06097 (unicellular cyanobacteria)	Dynamic assay (shear rate 40 s^−1^) Z8 medium 7.5 h and 6 weeks, 25 °C	− Lower biofilm development on epoxy-coated glass was observed than on glass.	[121]
**Combined strategy of chemically bioactive coatings and biocide-free coatings**				
**Surface/coatings**	**Organism**	**Experimental setup**	**Major findings**	**Reference**
Glass Smooth and patterned PDMS samples (biomimicking micropatterned surfaces inspired by the marine decapod crab *Myomenippe hardwickii*) coated with 1H,1H,2H,2H-perfluorododecyltrichlorosilane, zwitterionic polymer brush, which consists of sulfobetaine, and with layer-by-layer assembly of polyelectrolytes	*Amphora coffeaeformis* (diatom) *Amphibalanus* *amphitrite* (barnacle)	Static assay Filtered seawater Diatom adhesion (24 h, 24 °C) Barnacle settlement (48 h)	− Surface microtopography and sulfobetaine brushes − significantly affect diatom cellular adhesion − A synergistic effect when the microtopographies are combined with a zwitterionic polymer brush and with the assembly of polyelectrolyte coatings was observed in the barnacle settlement	[14]
Graphene–silicone rubber composite surfaces	*Paracoccus pantotrophus* (bacterium)	Artificial seawaterQuasi-static assay (7 days)Dynamic assay (7 days, from 0.2 to 0.5 m.s^−1^)	− Under dynamic conditions, graphene-based surfaces showed better antifouling performance when compared to results from the quasi-static assay	[104]
Graphene oxide–-silicone rubber composite surfaces	*Triceratium* sp. (diatom)	Static assay (8 days) Dynamic assay (10 days, 3.4 m.s^−1^) Algal broth medium	− Under hydrodynamic conditions, lighter colors and low Young’s moduli provided enhanced performance − Surfaces with 0.36 wt% of graphene oxide showed excellent antifouling performance	[150]
Irgarol^®^ 1051 * and Econea^®^ biocide ** immobilized to polyurethane and foul-release PDMS surfaces	*Pseudoalteromonas tunicate* (bacterium)	Väätänen Nine-Salt Solution medium 24 h	− Adhesion reduction higher than 90% for polyurethane formulations containing single biocides and close to 100% for PDMS with combined biocides	[41]
Nanomagnetite–hydroxyl-modified MWCNT–silicone-oil-infused PDMS coating	Marine bacteria	Fresh seawater 24 h and 30 days, 28 °C	− Coating presented antibiofilm adhesion performance (98% removal rate)	[101]
PDMS-based marine coating containing grafted Econea^®^ biocide ***	*Pseudoalteromonas tunicate* (bacterium)	Dynamic assay (shear rate 40 s^−1^) Väätänen Nine-Salt Solution medium 7 weeks, 25 °C	− Multifunctional coating showed antifouling effects after seven-week assays	[151]
Silicone-oil-infused CNTs/epoxy resin coating	*Chlorella* sp. *Phaeodactylum tricornutum* (algae)	Artificial seawater 21 days, 22 °C	− Coating demonstrated a greater inhibition of algae biofilm formation (up to 90% cell reduction)	[100]
Graphene oxide/silver nanoparticle–polypropylene sensor	*Halomonas pacifica* (bacterium)Marine microalgae	Static assayMarine broth 24 h, 26 °C Adam medium (artificial freshwater) 1 week	− Graphene oxide/silver nanocomposites showed more than 80% biofilm inhibition, as well as no visible fouling by microalgae	[15]
Fluorinated MWCNT-coated silicon surfaces	*Escherichia coli* (bacterium)	Phosphate-buffered saline, 6 h, 37 °C	− Incorporation of fluorinated MWCNTs decreased CFUs (about 98%)	[138]
Reduced graphene oxide/PDMS Graphene oxide–boehmite nanorod/PDMS composites	*Staphylococcus aureus* *Kocuria rhizophila* *Pseudomonas fluorescens* *Pseudomonas aeruginosa* (bacteria) *Candida albicans* (yeast) *Aspergillus brasiliensis* (fungus)	Nutrient-infused medium 3 weeks, 25 °C	− Boehmite nanorod composite coating showed higher antibacterial activity in comparison with bare PDMS and reduced graphene oxide/PDMS	[107]

Abbreviations: CFUs—colony-forming units, CLSM—confocal laser scanning microscopy, CNTs—carbon nanotubes, CS—chitosan, DEPs—differentially expressed proteins, IC50—median inhibition concentration, GNP—graphene nanoplatelet, LC50—half lethal concentration, MIC—minimum inhibitory concentration, MCE—methanol cell extract, MWCNTs—multi-walled carbon nanotubes, PDMAEMA—cationic polymer brush, PDMS—polydimethylsiloxane, PHEMA-co-PEG10MA—neutral polymer brush, PLA—Poly(lactic acid), PMMA—poly(methyl methacrylate), PSBMA—zwitterionic polymer brush, PSPMA—anionic polymer brush, PVC—polyvinyl chloride, wt%—weight percent. * Irgarol^®^ 1051 (N′-tert-butyl-N-cyclopropyl-6-(methylthio)-1,3,5-triazine-2,4-diamine). ** Econea^®^ biocide (4-bromo-2-(4-chlorophenyl)-5-(trifluoromethyl)-1H-pyrrole-3 carbonitrile). *** GBA26 (N-(2-aminoethyl)-3,4,5-trihydroxybenzamide hydrobromide).

#### 3.1.2. *In Situ* Studies

The marine environment is a complex habitat comprising up to 4000 potentially biofouling species [152]. Due to physiochemical intercommunication between different fouling species and all commensal, mutualistic, symbiotic, and additional relationships, *in situ* models can represent a more realistic approach than *in vitro* studies. Moreover, *in situ* marine biofilm studies allow the evaluation of biofilm properties under native conditions (undisrupted) and performing studies for a long time under natural conditions. Likewise, as commercial antifouling coatings should maintain antifouling capabilities for sometimes several years, *in situ* studies on natural marine environments may be particularly adequate [63]. Although there is no universal model for marine field tests, a minimum test period of six months is recommended since biofouling shows spatiotemporal variation under different seasons, temperatures, salinities, and light regimes [141], and limitations of the coatings will be revealed over a longer test period [64].

In turn, *in situ* studies usually require higher costs and specific equipment and devices related to the installation and sampling in natural marine environments. Moreover, sampling may be time-consuming and may be affected by natural conditions that, in some cases, are out of the control of the researchers, such as sea storms [153]. Most knowledge about biofouling and the performance of antifouling coatings has been conducted in the laboratory or *in situ*, in wave-protected habitats, usually in bays and port harbors. One of the main drawbacks related to *in situ* tests is the scarcity of studies performed under high-energy environmental conditions, such as under moderate and strong wave and current activity, due to logistical and safety-related difficulties in conducting detailed observations [153,154,155]. Since these high wave-energy regions of coastal oceans are becoming increasingly targeted as areas of human activity, such as aquaculture, and as a source of renewable energy, it is critical to improve knowledge about biofouling risks in these environments, as well as the evaluation of novel antifouling surfaces that can be used in the material design of relevant industrial equipment.

Table 3 shows *in situ* studies focused on different marine surfaces/coatings developed for marine environments. Most *in situ* tests of novel marine coatings are performed after *in vitro* analyses to confirm if the effectiveness obtained under laboratory conditions is equivalent to what was achieved in natural marine environments [14,41,81,89,94,102,106,107,108,137,145,151]. Although, in most cases, similar results are obtained between both tests [94,102,137,151], some contradictory findings have also been reported [14,89]. An *in vitro* study performed to test biomimicking micropatterned surfaces concluded that the settlement of barnacles on the patterned and smooth surfaces was similar [14]. However, in the field tests in natural seawater, barnacle settlement on the smooth surface was detected after 4 weeks of immersion, while no barnacles were observed on the patterned surfaces during the 7 weeks of the immersion period. Since it has been demonstrated that the antifouling properties of micropatterned surfaces may be associated with hydrodynamic forces, and the hydrodynamic conditions between the static laboratory and field tests were different, this may have contributed to the differences found [61,63]. Moreover, the discrepant period between *in vitro* (hours/days) and *in situ* (weeks/months) tests can also affect the performance of antifouling coatings [14,89]. Likewise, a study performed with pristine silicon rubber, graphene-added silicon rubber, and graphene-added silicon rubber filled with quaternary ammonium salt showed that the bactericidal graphene-added silicon rubber filled with quaternary ammonium salt coating exhibited an anti-adhesion effect under laboratory conditions, but the anti-adhesion effect was not durable since it lost antifouling effects completely in real marine conditions [106].

Unfortunately, few studies conduct a more realistic assessment of the performance of novel coatings due to the costs involved in the process [41]. After the determination of the minimal inhibitory concentrations (MICs) and minimal bactericidal concentrations (MBCs) of two commercial and functional biocides and the *in vitro* evaluation of biofilm adhesion potential of a marine bacterium on the surfaces with the immobilized biocides, *in situ* analyses were performed for up to 66 weeks in two different marine environments (Portugal and Singapore) [41]. Additionally, trial field tests on two coated ships with these formulations were accomplished. The ships traveled around the world (including Brazil, Cape Verde, and Greenland), experienced distinct ecosystems, and were also subjected to periodic dock stages. The analyses were performed after the ships had been traveling between eight and fourteen months and reflected the previous *in situ* results, corroborating biofilm adhesion performance, which demonstrates the predictive power of *in situ* testing [41].

**Table 3:** *In situ* studies focused on different surfaces/coatings used in marine environments in the last years. The different surfaces/coatings were divided into non-modified surfaces, chemically bioactive coatings, and a combined strategy of chemically bioactive coatings and biocide-free coatings. The distribution by rows follows a chronological order.

**Non-Modified Surfaces**				
**Surface/Coatings**	**Organism**	**Experimental Setup**	**Major Findings**	**Reference**
Titanium Aluminum Limestone Shale Glass	Deep-sea bacterial communities	Ionian Sea, Greece Sea (1500, 2500, 3500, and 4500 m below the water level and surfaces were deployed in vertical and horizontal positions) 155 days, 14 °C	− Depth played an important role in the composition of deep-sea biofouling communities, while substratum type and the orientation of substrata throughout the water column were less important	[156]
**Chemically bioactive coatings**				
**Surface/coatings**	**Organism**	**Experimental setup**	**Major findings**	**Reference**
Polystyrene Teflon^®^ Sea Quantum Classic^®^ antifouling commercial coating (Cu_2_O–CuPy (copper(I) oxide–copper pyrithione)) Intersmooth^®^ 360 antifouling commercial coating (Cu_2_O–ZnPy (copper(I) oxide–zinc pyrithione) 2 antifouling coatings synthesized at the laboratory (Cu_2_O (copper(I) oxide)–A4S ^®^–Sea Nine ^®^–Zineb ^®^ and Cu_2_O (copper(I) oxide)–Zineb ^®^)	Microfoulers	French Mediterranean coast (Toulon military harbor and the natural protected area of Porquerolles Island) 2 weeks	− Pioneer microalgal communities on all surfaces were dominated by the same two diatom species: *Licmophora gracilis* and *Cylindrotheca closterium* − A low diatom abundance was observed on antifouling coatings when compared to polystyrene and Teflon^®^	[157]
Graphene oxide in alkyd resin surface	Micro- and macrofoulers	Lagoon with tidal water directly connected to Jeju Sea, South Korea3 weeks	− Graphene-oxide-coated surfaces greatly reduced biofouling	[137]
Cationic polymer brush (PDMAEMA) Anionic polymer brush (PSPMA) Neutral polymer brush (PHEMA-co-PEG10MA) Zwitterionic polymer brush (PSBMA)	Micro- and macrofoulers	Hartlepool Marina, County Durham, UK (50 cm below the water level) 2 months, 12–15 °C	− PSPMA and PDMAEMA had better antifouling properties than PHEMA-co-PEG10MA and PSBMA after one week of immersion − After eight weeks, no significant differences in biofouling coverage were observed among the surfaces	[89]
Glass PDMS MWCNT–PDMS surfaces Titanium dioxide–PDMS surfaces	Bacteria and diatoms	Natural seawater, Zhoushan, China (0.5–1 m below water level) 28 days	− MWCNT and titanium dioxide–PDMS surfaces improved bacterial density, but MWCNT–PDMS surfaces decreased diatom density in biofilms after 28 days	[108]
Self-repairing coating (PDMS -based polyurea (PDMS–PUa) with a small amount of organic antifoulant (4,5-dichloro-2-noctyl-4-isothiazolin-3-one)	Micro- and macrofoulers	Xiamen Bay, China (1 m below the water level) 6 months	− The coating has excellent antifouling/fouling-release performance, and it completely recovered its mechanical properties after damage	[158]
Cupreous coatings Primocon ^TM^ (commercially available paint)	Micro- and macrofoulers	Auckland Westhaven Marina, New Zealand 3 months	− With increasing copper concentration, bacterial diversity decreased while eukaryotic diversity increased − The highest copper concentration promoted a less taxonomically diverse microcommunity	[159]
Carboxyl- and hydroxyl-modified MWCNT–PDMS nanocomposites	Micro- and macrofoulers	Weihai Western Port, China (1.5 m below the water level) 56 days, 11 °C	− Carboxyl-modified MWCNT–PDMS nanocomposite with lower carboxyl content % (*w/w*) demonstrated a strong perturbation effect on pioneer prokaryotic colonization	[160]
Graphene oxide–alumina nanorod–PDMS nanocomposites	Micro- and macrofoulers	Natural seawater 3 months pH of 7.6–8.3, salinity of 37%, 23–28 °C	− No fouling or surface deterioration for the nano-filled sample was observed, as opposed to pristine PDMS	[94]
Hydroxyl-modified MWCNT–silicone-oil-infused PDMS coatings	Marine bacteriaMicro- and macrofoulers	Yellow Sea, China (1–2 m below the water level) 8 months	− Anti-adhesion and antifouling properties were enhanced when higher volume ratios of hydroxylated MWCNTs were used	[102]
Graphene oxide–cuprous oxide nanoparticle-coated acrylic resin surfaces	Micro- and macrofoulers	South China Sea (0.2–2.0 m below the water level, weak water currents, less than 2 m.s^−1^) 90 and 365 days	− Bare panels showed an abundant growth of marine organisms within 90 days, while coated surfaces were hardly fouled by marine organisms after 365 days	[161]
Acrylic (Plexiglass^®^) Ceramic tile Acrylic covered with Safety Walk^®^ (a non-slip surface)	Micro- and macrofoulers	Cartagena Bay, Chile (a fully exposed bay to the predominant incoming waves propagating, 5 and 15 m below the water level, 11–17 °C) Up to 23 months	− Ceramic tiles achieved higher biomass than the other materials, but differences also varied with depth and month of the year − In all materials, surface cover reached 100% within 1 month at 5 m deep in spring and summer months, and over 70% at 15 m deep, with lower cover in winter months	[153]
Aluminum High-density polyethylene Steel Copper-based antifouling paint based on high solid epoxy polyamine (with micaceous iron oxide)	Macrofoulers	Cartagena Bay, Chile (a fully exposed bay to the predominant incoming waves propagating, 5 and 15 m below the water level, 11–17 °C) Up to 7 months	− All materials were colonized within 3 months, with no significant differences in species composition, total cover, or the rate of biomass accumulation − No significant attachment was found on plates coated with the antifouling paint after 7 months of exposure	[155]
Carboxyl- and hydroxyl-modified MWCNTs Graphitized MWCNTs Carboxyl-modified single-walled carbon nanotube (SWCNT)–PDMS surfaces	Pioneer biofilm bacteria	Xiaoshi Island, China (1.5 m below the water level) 24 days, 10–17 ° C	− All carbon nanotube (CNT)–PDMS surfaces reduced Proteobacteria biofilm formation but increased cyanobacteria biofilm development	[162]
Copper-based self-polishing-based fiberglass antifouling coatings Uncoated fiberglass Nylon ropes	Marine filamentous fungi	Marina Bandar Rowdha, Sea of Oman (1 m below the water level) 6 months, 24–30 ° C, pH of 8.2	− Six fungal isolates were obtained from the antifouling coating, while just four isolates were isolated from the uncoated fiberglass and nylon ropes − Fungi isolated from the antifouling coating were highly resistant to copper	[163]
Methanol cell extract (MCE) from *Bacillus licheniformis* incorporated in a self-polishing paint at 2 and 5% *w*/*w*	Micro- and macrofoulers	Gulf of Aqaba, northern Red Sea (6–8 m below the water level) 6 months	− Fouling coverage was reduced by 30% in the 5% MCE-treated panels in comparison with the control panels	[81]
Elasnin-based coatings	Micro- and macrofoulers	Fish farm at Yung Shue O, Hong Kong 4 weeks	− Elasnin-based coatings inhibited the formation of multi-species biofilms and the attachment of large biofouling organisms − Coatings began to lose their effectiveness after the third week in the field	[164]
Graphene hydroxide/ silver composite, bare graphene oxide, and MWCNTs incorporated into PDMS-based coatings	Micro- and macrofoulers	Persian Gulf, Iran (1 m below the water level, 23–32 °C, pH of 8, salinity of 38 ppm, oxygen of 6 mg.L^−1^) 60 days	− 0.5 wt% graphene hydroxide/silver composite coating showed better performance in preventing biofilm formation and the attachment of fouling organisms	[98]
Pristine silicon rubber Graphene-added silicon rubber Graphene-added silicon rubber filled with quaternary ammonium salt coatings	Micro- and macrofoulers	East China Sea 9 months	− Anti-adhesion effect of graphene-added silicon rubber filled with quaternary ammonium salt coating was not durable − The non-bactericidal film of the graphene-added silicon rubber coating exhibited stronger antifouling ability when immersed in the marine environment for 9 months	[106]
Guanidine-functionalized graphene/boron acrylate polymer composite	Micro- and macrofoulers	Yellow Sea, China 2 months	− Field trials revealed no fouling adhesion or surface deterioration	[145]
PVC coated with 3 different environmentally friendly alkaloids (5-chlorosclerotiamide, circumdatin F, and notoamide C)	Micro- and macrofoulers	Fish farm in Daya Bay, China (1 m below the water level) 30 days	− The three alkaloids affected the composition and diversity of marine-fouling microbial communities − The 5-chlorosclerotiamide and notoamide C coated PVC completely inhibited many macrofouler-inductive bacteria	[165]
Acrylic-acid-modified graphene oxide/acrylate composites	Micro- and macrofoulers	Zhoushan Sea, China 6 months	− The composites exhibited self-polishing antifouling performance and high corrosion resistance and mechanical strength	[166]
CNT CNT coated with cyanoacrylate (polymer coating)	Micro- and macrofoulers	Atlantic Ocean, off the eastern coast of Florida 12 months	− Cyanoacrylate coatings increase durability and reduce the colonization of biofouling compared to CNTs	[167]
Albofungin-based antifouling coatings	Micro- and macrofoulers	Fish farm at Yung Shue O, Hong Kong (0.5 m below the water level) 2 months	− During 12 days of observations, the biofilm quickly grew on the control surfaces and consisted of diverse microorganisms, whereas the diversity of microorganisms on the surface covered with 5 wt% of albofungin-based coating reduced significantly	[72]
Acrylic (Plexiglass^®^) Steel Stainless steel Micanti (modified substrate surface coating: a nylon fiber and polyester film with a furry texture and a two-component water-based adhesive) Seavoyage 100 CDP Sherwin-Williams (CDP, a copper-based slow-release controlled-wear polymer antifouling paint) Seavoyage A/F-21 Sherwin-Williams (F21) copper-based antifouling paint Ocean Jet 33 (OJ33) copper-based antifouling paint	Micro- and macrofoulers	Cartagena Bay, Chile (a fully exposed bay to the predominant incoming waves propagating, 5 and 15 m below the water level, 11–17 °C) Up to 15 months	− The two traditional copper-based antifouling coatings and the slow-release antifouling paint showed similarly low biomass and richness, indicating their efficacy after 12 months of exposure	[154]
Chitosan–melanin hybrid nanoparticle coatings (0.5, 1, 2 and 3 wt%)	Micro- and macrofoulers	Persian Gulf, Bandar Abbas, Iran (1 m below the water level) 3 months	− The most effective results were observed for the coatings with 3 wt% chitosan–melanin hybrid nanoparticles.	[85]
**Combined strategy of chemically bioactive coatings and biocide-free coatings**				
**Surface/coatings**	**Organism**	**Experimental setup**	**Major findings**	**Reference**
Smooth and patterned PDMS samples (biomimicking micropatterned surfaces inspired by the marine decapod crab *Myomenippe hardwickii*) coated with 1H,1H,2H,2H-perfluorododecyltrichlorosilane	Micro- and macrofoulers	Natural seawater (0.5 m below the water level) 7 weeks	− More organisms settled on the smooth than on the patterned surfaces − Barnacle settlement on the smooth surface was detected after 4 weeks of immersion − No barnacles were observed on the patterned surfaces during the 7 weeks	[14]
PVC Intersleek 757 (biocide-free commercial coating) 5 self-polishing copolymer coatings (Cu_2_O–CuPy (ZnO) *, Cu_2_O–ZnPy, Econea–Sea Nine, Cu_2_O–A4S–Sea Nine–Zineb (ZnO) *, and Cu_2_O–CuSCN–ZnPy)	Microfoulers	Toulon Bay, northwestern Mediterranean Sea, France (1 m below the water level) 30 days	− Biocide-free coatings showed higher densities than all other coatings, except for one biocidal coating − Heterotrophic bacteria showed the highest densities, and diatoms showed the lowest, but the relative abundances of these groups varied depending on the coating − Copper-free self-polishing copolymer coatings failed to prevent diatom settlement	[79]
Irgarol^®^ 1051 ** and Econea^®^ biocide *** immobilized to polyurethane and foul-release PDMS surfaces	Micro- and macrofoulers	Estaleiros Navais de Peniche dock, Portugal (3 m below the water level, pH of 8.3, 14–22 °C) Raft in Singapore (3 m below the water level, and temperature ranged between 32 and 35 °C) 66 weeks	− For the foul-release PDMS surface, antifouling performance only started to show differences among the formulations after 45 weeks of exposure − The formulation containing both immobilized biocides exhibited better antifouling performance	[41]
PDMS-based marine coating containing grafted Econea^®^ biocide ***	Micro- and macrofoulers	Estaleiros Navais de Peniche Dock, Portugal (3 m below the water level, pH of 8.3, 13–22 °C) 30 months	− After 23 weeks some slime occurred on the control surface − The multifunctional coating showed auspicious antifouling effects	[151]
PVC Copper-releasing coating PDMS-based elastomer FRC	Heterotrophic prokaryotes	Toulon Bay and Banyuls-sur-Mer Bay (lower nutrients and stronger currents than Toulon Bay), Mediterranean coast, France 75 days	− Low and unique diversity was found in the copper-releasing coating − Differences were found between the two locations since the biofilm developed in Banyuls Bay was less dense compared to those formed in Toulon and presented a slower biofilm formation	[168]
Reduced graphene oxide/PDMS Graphene oxide-boehmite nanorod/PDMS composites	Micro- and macrofoulers	Tropical area 45 days, 23–28 °C	− The higher self-cleaning and foul-release performance of the boehmite nanorod composite coating was observed	[107]

Abbreviations: CNTs—carbon nanotubes, Cu_2_O—copper oxide, CuPy—copper pyrithione, CuSCN—copper thiocyanate, FRCs—fouling-release coatings, MCE—methanol cell extract, MWCNTs—multi-walled carbon nanotubes, PDMAEMA—cationic polymer brush, PDMS—polydimethylsiloxane, PHEMA-co-PEG10MA—neutral polymer brush, PSBMA—zwitterionic polymer brush, PSPMA—anionic polymer brush, PVC—polyvinyl chloride, SWCNTs—single-walled carbon nanotubes, wt%—weight percent, ZnO—zinc oxide, ZnPy—zinc pyrithione. * Zinc oxide (ZnO) is not considered a biocide by regulations but was added in the formulation. ** Irgarol^®^ 1051 (N′-tert-butyl-N-cyclopropyl-6-(methylthio)-1,3,5-triazine-2,4-diamine). *** Econea^®^ biocide (4-bromo-2-(4-chlorophenyl)-5-(trifluoromethyl)-1H-pyrrole-3 carbonitrile).

Some *in vitro* models have evolved to mimic the real conditions in marine environments. However, *in situ* studies in real marine environments allow for long-time and surface tribological characterization, but they are also more complex. Overall, the main advantages and limitations of *in vitro* and *in situ* tests are described in Table 4.

## 4. Concluding Remarks

To date, there is no available universal strategy that is effective against marine biofouling. Compared to chemical treatment agents, fewer toxicological and environmental risks are often associated with non-chemical treatment agents. Successful solutions can be implemented from the combination of different strategies, such as the use of wipers with chemical compounds, which provide both physical and biological protection, or by the incorporation of UV radiation on a non-stick foul-release or self-polishing coating to match the performance of existing systems at reduced costs [45].

The improvement of environmentally friendly marine coatings such as protein-resistant polymers, FRCs, and bioinspired antifouling coatings is crucial for improved antifouling strategies. Advances in genetic tools may also provide a better understanding of the molecular mechanisms and biofilm-related functions [123,124,125], creating a high-throughput screening approach to find new targets for disrupting biofilms. In the progress of novel antifouling coatings, factors related to production, application, maintenance, and service life should be considered. Novel promising marine coatings should be non-toxic, effective in a wide range of applications, require low maintenance, have reduced cost, and maintain high performance over long periods [61]. Among chemically active antifouling technologies, SPC coatings are the most promising antifouling technology due to their long-term efficiency in service life. In turn, from the biocide-free coating approaches, FRCs are the most suitable for the marine industry due to their eco-friendly biocide-free attributes.

The heterogeneity and structural complexity of marine biofilms pose a great challenge to their evaluation and control. The initial *in vitro* screening of promising, novel coatings is an important step for selecting those that will be further tested *in situ*. Reliable *in vitro* models must strive to reproduce the environmental conditions present in marine systems, as these factors affect the biofilm structure, composition, and mechanical properties. While *in vitro* models are powerful tools for reproducibly testing the efficacy of different coatings and controlling some environmental parameters simultaneously, they fail to account for the complex and dynamic nature of the interactions that play out between marine organisms. Even though there is no gold-standard *in vitro* model for the study of marine biofouling, it is crucial to know the limitations of selected models so as to not over-extrapolate data and produce assumptions beyond the abilities of the model. A promising approach is to use *in vitro* testing using defined conditions that are relevant to the environmental scenario that is being mimicked (including the use of relevant organisms, media, hydrodynamics, etc.) as a screening tool and then proceed to *in situ* studies (by immersion) over extended periods to confirm the screening results. Further validation tests should include exposure to the actual working environment (for instance, using panels in a ship hull during its routine operation) to include the variability in shear conditions (docking and sailing periods) and the change in environmental conditions imposed by the geographical diversity found during the operation.

Overall, investing in the research and development of innovative technology that can provide practical and feasible tools to control biofouling while protecting the marine environment from harmful chemical and/or biological waste is essential. Therefore, economic factors and biosecurity risk-management decisions should be taken into consideration to contemplate the practicality, feasibility, and environmental impact of biofouling management options.

## Figures and Tables

**Table 4:** Main advantages and limitations of *in vitro* and *in situ* studies.

	Advantages	Limitations
***In vitro*** **studies**	Simplicity, speed, and low cost	Missing interactions between different marine organisms
	Precise control of experimental parameters	Nutrient availability differs from the natural environment
	Customizable, controllable, and reusable methodology	Direct real-time monitoring is not always an option
	Design flexibility, allowing the study of the effects of single elements on various aspects of biofilm development	Lack of all changes associated with environmental parameters that occur in real aquatic environments (e.g., pH, temperature, and hydrodynamic condition variations)
***In situ*** **studies**	Allow the study of complex interactions between marine organisms	More expensive
	Resemble natural marine conditions	Sampling limitations by natural conditions
	Allow the study of higher hydrodynamic conditions such as those found under high-energy environments.	Time-consuming sampling
	Studies can be performed for a long time (months/years) and enable surface tribological characterization upon long immersion periods (friction coefficient, wear, temperature, durability of surfaces under harsh marine environments).	Requirement of specific equipment, devices, and specialized personnel related to the installation and sampling

## Data Availability

Not applicable.
